# Manipulated taking the agent versus the recipient perspective seems not to affect the relationship between agency-communion and self-esteem: A small-scale meta-analysis

**DOI:** 10.1371/journal.pone.0213183

**Published:** 2019-02-28

**Authors:** Olga Bialobrzeska, Michal Parzuchowski, Bogdan Wojciszke

**Affiliations:** 1 Department of Psychology, SWPS University of Social Sciences and Humanities, Warsaw, Poland; 2 Center of Research on Cognition and Behavior, Faculty in Sopot, SWPS University of Social Sciences and Humanities, Sopot, Poland; Mälardalen University, SWEDEN

## Abstract

There is a growing debate about the relationship between self-perceived agency-communion and self-esteem. One viewpoint for this debate is offered by the Dual Perspective Model, a novel theoretical framework that introduces the agent and the recipient as two fundamental perspectives in social perception. Building on this model, we expected higher importance of self-ascribed agency for self-esteem in the agent perspective than in the recipient perspective and a higher importance of self-ascribed communion for self-esteem in the recipient than in the agent perspective. However, the meta-analysis of six experiments (*N* = 659, 68% females) showed no interaction of the perspectives and self-ascribed agency and communion in predicting self-esteem. These findings demonstrate that the relationship between agency-communion and self-esteem seems to be fairly independent of one’s temporary mindset.

## Introduction

There is strong evidence in the published literature that self-esteem is determined by how one evaluates oneself on the agency dimension [1–3]. The role of self-ascribed communion is more ambiguous, though. Some researchers argue that the belief about one’s communion is weakly related to self-esteem [3], whereas others claim that communion (and especially morality) is essential for self-concept [4–6]. There is still not much research on the moderators of the link between agency-communion and self-esteem. In the present paper we examine one such moderator–taking the agent versus the recipient perspective, which is based on a new theoretical framework, the Dual Perspective Model by Abele and Wojciszke [1,7]. This extends our recent work which documented that taking the agent perspective moderates the relationship between self-ascribed agency and self-esteem [8]. The current article addresses the same issue but this time we used an experimental and behavioral manipulation [9] of the agent versus recipient perspective, rather than measure it as an individual difference (a habitual preference to take an action and influence others).

### Agency and self-esteem

Numerous studies have indicated that the primary basis of self-esteem is the effectiveness of one’s actions. The relationship between self-esteem and achievements has already been discussed in William James’s works [10]. James described self-esteem as a ratio of achievements and aspirations. Further studies have repeatedly confirmed James’s idea, showing that self-esteem is affected by achievements in personally important life domains [11–15].

The agency-over-communion effect in self-esteem was shown in the experimental research, in which belief in one’s agency and communion were manipulated and self-esteem was subsequently assessed [16]. Priming participants with positive and negative information concerning their agency had a significant effect on their self-esteem, while the information concerning their communion did not.

Similarly, in a series of correlational studies, Wojciszke et al. [3] demonstrated that self-esteem is strongly related to how people view their own agency and, to a lesser extent, to how they view their own communion. Self-ascribed agency was a strong predictor of self-esteem among both men and women of various ages, as well as among those who believe they value agency more than communion and use it as the base for their self-esteem as well as those who believe the opposite. Yet, self-ascribed communion was a much weaker predictor than self-ascribed agency or did not predict self-esteem at all. This could be alternatively explained by the fact that the self-esteem scales are associated with agency; however, the same phenomenon was demonstrated when self-esteem was measured with self-liking and self-competence [17] subscales separately or with a non-declarative and implicit measure [3].

### Communion and self-esteem

On the other hand, some researchers argue that communion plays a key role in self-esteem. For example, according to sociometer theory [5], self-esteem is an indicator of being accepted by others; therefore self-esteem has to be highly related to how good (communal) a member of the community one is. However, Mahadevan, Gregg, Sedkides and de Waal-Andrews [18] examined two potential functions of self-esteem: tracking an individual level of social acceptance (*sociometer theory*) and tracking agency-based status in the social hierarchy (*hierometer theory*), and found stronger support for the latter.

Yet, recent research has suggested that the moral facet of the communion dimension could be of particular importance for shaping self-view. A study by Abele et al. [19] showed that self-esteem was related to morality, but not to the warmth facet of communion. Similarly, but on the group level, Leach, Ellemers and Barreto [20] found that in-group morality affected positive in-group evaluation, while in-group sociability and competence were less important for in-group identification.

In overall, the role of agency for self-esteem appears to be a more solid and universal phenomenon, whereas the role of communion forms a more complex picture that is relevant only for some societies [19,21,22] or when measured with one of communion’s facets [19]. Studying the possible moderators of the agency-communion relation to self-esteem is one of the key issues in this area of research.

In the present research we sought to test a situational determinant of when people construe their self-worth more in terms of self-ascribed agency or communion. According to the Dual Perspective Model, one such determinant could be taking the agent versus recipient perspective. As we were interested in a temporary, situational taking of the agent and the recipient perspectives, we explored this issue in an experimental paradigm by manipulating the perspectives. To the best of our knowledge the present research is a first attempt to experimentally test how assuming mindsets (agent and recipient perspective) can moderate the agency-communion effect on self-esteem.

### Dual Perspective Model

The Dual Perspective Model proposed by Abele and Wojciszke [1,3,7,8] originated from the simple observation that a fundamental feature of any social interaction is the presence of two dynamically changing perspectives, those of the agent and the recipient. The perspective of the agent is taken by the one who performs an action and has control over the situation (for example a speaker, driver, doctor), whereas the perspective of the recipient is taken by the one who experiences the consequences of an agent’s actions (correspondingly, a listener, passenger, patient).

Although a vast amount of research involves two distinct perspectives in the psychological literature (speaker–listener [23,24]; perpetrator–victim [25,26]; actor–observer [27]; for review see Malle [28]; help provider–recipient [29]; leader–follower [30–32]; moral agent–moral patient [33]; agent–actor [34,35]), Dual Perspective Model is rather a meta-dichotomy that describes the common thread of dichotomies that are present in the above distinctions’ literature and allows for predicting common consequences of taking the two perspectives.

The perspective of the agent is defined as taking an action and having control over a situation, whereas the perspective of the recipient is defined as experiencing others’ actions. In the agent perspective, the focus is directed toward the performance of an action, whereas in the recipient perspective, one focuses on what affects oneself, such as other people or certain sensations.

### Dual Perspective Model and agency-communion

The foundation of the Dual Perspective Model is research on agency and communion as two basic dimensions in social perception (for a review, see Abele & Wojciszke [7]). The association between agent-recipient perspectives and agency-communion arises from the functional nature of each perspective. In the agent perspective, that is, when one is performing an action, the current goal is to complete an action, and one must monitor the performance effectiveness. Therefore, taking the agent perspective entails the agency dimension. In the recipient perspective, that is, when one focuses on the stimuli that affect him or her, one must monitor the social value of others’ performance and their intentions. Therefore, taking the recipient perspective entails communion.

A key point for our research is that the functional value of agency in the agent perspective and communion in the recipient perspective is also relevant for self-perception. In the recipient perspective, that is, when being subjected to the actions of others, monitoring one’s own communion makes it more likely that one will be treated well and included in the group. It might prevent the recipient from being cold or immoral, which will likely result in harm and social exclusion (see Leary [5]). In the agent perspective, that is, when taking an action, the activation and use of agentic categories would be functional, as it allows the effectiveness of one’s own performance to be monitored and failure to be prevented. Therefore, a sense of self-worth could be more strongly related to communion in the recipient than in the agent perspective, and at the same time more strongly related to agency in the agent than in the recipient perspective [36].

To simplify, the Dual Perspective Model predicts that by taking the agent perspective, a manager would tend to see the world through the lenses of agency (efficiency, competences, determination) and use these categories in the social perception of both self and others, whereas the subordinate, by taking the recipient perspective, would tend to see the world through the lenses of communion (kindness, friendliness, fairness) and use these categories in the social perception of both self and others.

That is, the Dual Perspective Model allows for the prediction that a well-established agency-over-communion effect in self-esteem could be modified by taking the agent and the recipient perspectives. This does not necessarily mean that the agency-over-communion pattern would be reversed after taking the recipient perspective but that the effect of self-ascribed communion on self-esteem would be increased in the recipient compared to the agent perspective while retaining a high importance or even dominance of self-ascribed agency.

### Hypotheses

In order to investigate the rationale of the Dual Perspective Model in the context of self-perception, we tested if taking the agent-recipient perspectives moderates the relationship between self-ascribed agency-communion and self-esteem. First, we predicted that in the agent perspective, self-ascribed agency is more strongly related to self-esteem than in the recipient perspective and second, that in the recipient perspective, self-ascribed communion is more strongly related to self-esteem than in the agent perspective.

### Overview

All of the experiments had a between-subjects design and similar procedure. It started with the manipulation of taking the agent versus the recipient perspective, followed by the assessments of self-esteem, self-ascribed agentic and communal traits, and a manipulation check.

To operationalize the agent and recipient perspectives, we followed the principle that taking the agent perspective occurs when one is performing an action and/or focusing on action performance, whereas taking the recipient perspective occurs when one is a subject of someone else’s action and/or focusing on the impact of someone or something on oneself. We also attempted to use manipulations that were not directly related to power or status because we sought to capture ‘pure’ perspectives and not the other constructs. Overall, we used six different manipulations that were based on behaviors, episodic recall and priming.

Self-esteem was assessed as a state (feelings of self-worth [37]) with three different tools: the State Self-Esteem Scale by Heatherton and Polivy [38], Rosenberg’s Self-Esteem Scale [39] that was modified such that the items referred to temporary, not stable, attitudes toward oneself, and a single-item self-esteem scale (e.g., [40]). Self-ascribed agency and communion were assessed with a list of agentic and communal traits balanced for favorability and their agency and communion relatedness [41]). In three of the experiments we used an extended list of agentic and communal traits which distinguishes a sociability and morality subscales within a communion dimension. In two experiments, we introduced potential moderators of the effect of the agent-recipient perspectives on self-esteem, specifically, the status of the agent and recipient as well as the valence of the action that was performed or to which one was subjected.

As for the statistical analysis, we tested the moderation model (Model 1 [42]) in which the agent-recipient perspective was tested as a moderator (M) of the relationship between self-ascribed agency-communion (X) and self-esteem (Y). In two experiments, which included the second moderator, we tested the moderated moderation model (Model 3 [42]). For the sake of readability, the table with the descriptive statistics and tests of differences between conditions can only be found in S1 Table.

The sample size for each study was determined beforehand based on the typical sample size in experimental studies in social cognition literature at that time. Our studies were conducted in 2013–2014, when performing the power analysis in advance was not a standard procedure as it is now, and we followed the common rule of a minimum of 20–30 participants per condition. In Studies 1–3, after we reached the minimal number of 25 participants per condition, we continued the data collection till the end of the working day. In Studies 4–6, in which we expected smaller effect sizes because of more subtle manipulations (priming), we planned bigger sample sizes: min. 30 participants per condition in Study 4, min. 100 participants per condition in Study 5, and min. 50 participants per condition in Study 6 (the exact sample size was planned in consideration of the practical and financial constraints). Again, after reaching the planned sample size, we continued the data collection till the end of the working day. We are well aware that according to current standards the sample sizes of some of the present studies are too small. To directly address this problem we have integrated the results of all six of the experiments within a meta-analysis comprising 659 participants, which should allow the detection of a small-sized effect for at 80% power.

The present line of studies was conducted in accordance with the principle of conceptual replication [43–45]. To address the same research question, we applied multiple ways of operationalizing the independent variable as well as multiple assessments of the dependent variables. Employing a priori theoretical hypotheses, while including multiple studies, and conceptual replications, prevented the inflation of false-positive rates [46]. All data and materials used in all studies are available via OSF at https://osf.io/ag8sk/.

## Experiment 1

In Experiment 1, we examined whether the agent and the recipient perspectives, manipulated by playing the assigned role in the situation arranged in the lab, affected the importance of agency and communion for self-esteem.

### Method

#### Participants

The participants were fifty-two undergraduate native Polish students from SWPS University of Social Sciences and Humanities in Sopot (*M*_age_ = 21, *SD* = 2.25, 39 females, 12 males, and one person who did not specify sex).

#### Manipulation

The participants took part in the experiment in pairs. As the result of a draw, they learned that one of them (agent condition) was supposed to plan and note one weekend day for the second participant. During this task, the participant could use the leaflets of various city attractions (e.g., walking tours, restaurants, cinema), and they could come up with their own ideas. At the time when one participant was occupied with scheduling the day for the second participant, the other one (recipient condition) only watched. They were not allowed to communicate.

We expected that planning the day for another person would result in taking the agent perspective because one performs and focuses on an action over which one has control. Conversely, we expected that being a person for whom the day was being planned and merely observing such planning would result in taking the recipient perspective because the actions were directed at a person who had no control over them.

#### Procedure

The design was between subjects. After the manipulation, the participants completed the scales. To assess self-esteem, we used the State Self-Esteem Scale by Heatherton & Polivy ([38]; e.g., *I feel good about myself*, *I am pleased with my appearance right now*; a five-point scale that ranges from 1 [*not at all*] to 5 [*extremely*]; *α* = .85). The self-ascriptions of agentic and communal traits were assessed with eight agentic traits (e.g., *ambitious*, *self-confident*, *determined; α* = .83) and eight communal traits (e.g., *friendly*, *helpful*, *sensitive to others; α* = .88 [41]). The participants indicated the extent to which each trait could be used to describe them on a scale from 1 (*definitely not*) to 7 (*definitely yes*). The manipulation check was assessed with four items (*During this study I had a sense of influence*; *I had a little control over what was happening; In this study*, *I was reliant on what the other participant was doing*; *My role in this study could be described as passive;* answered on a scale from 1 [*definitely not]*, to 5 [*definitely yes*]; *α* = .84). At the end, the participants also reported their age and gender.

### Results

The analysis of the manipulation check items indicated that the manipulation was successful. The participants in the agent perspective condition agreed with the statements that during the study they had a sense of influence and control over the situation, that they were not reliant on the other participant, and that they were not passive to a higher extent than the participants in the recipient perspective condition, *t*(50) = 17.68, *p* = .000, *d* = 5.00 (one-sided).

The analysis of Hypothesis 1 about the moderating effect of the agent-recipient perspective on the relationship between self-ascribed agency and self-esteem revealed no interaction effect, *b* = -0.06, 95% CI = [-0.31, 0.20], *t*(48) = 0.45, *p* = .653. This indicates that the strength of the relationship between self-ascribed agentic traits and self-esteem was not significantly different among the participants in the agent condition, *b* = 0.20, 95% CI = [-0.02, 0.42], *t*(48) = 1.86, *p* = .069 and the participants in the recipient condition, *b* = 0.26, 95% CI = [0.13, 0.39], *t*(48) = 3.99, *p* = .000.

The analogous model was tested for self-ascribed communal traits as a predictor variable. Again, contrary to our hypothesis, the interaction was not significant, *b* = -0.00, 95% CI = [-0.25, 0.25], *t*(48) = 0.01, *p* = .993. Self-ascribed communion was significantly related to self-esteem both among the participants in the agent condition, *b* = 0.21, 95% CI = [0.02, 0.40], *t*(48) = 2.17, *p* = . 035, and the participants in the recipient condition, *b* = 0.21, 95% CI = [0.04, 0.37], *t*(48) = 2.56, *p* = .014.

In summary, we did not find an expected interaction effect of self-ascribed agency-communion and the agent-recipient perspectives on self-esteem. Both self-ascribed agency and self-ascribed communion were positively related to self-esteem, and the strength of the relationship did not differ in the agent and the recipient perspective.

## Experiment 2

In Experiment 2, our aim was to examine the interaction effect between self-ascribed agency-communion and the agent-recipient perspectives on self-esteem with a different manipulation of the agent-recipient perspectives. The dependent variables, that is, self-esteem and self-ratings on agentic and communal traits, were measured in exactly the same way as in Experiment 1.

### Method

#### Participants

The participants were forty-eight undergraduate native Polish students from Gdansk University of Technology (*M*_age_ = 22.32, *SD* = 1.37, 24 females, 21 males and 3 persons who did not specify sex). One participant who failed to properly complete the questionnaire was excluded from analysis.

#### Manipulation

As in Experiment 1, the participants took part in the experiment in pairs and were randomly assigned to the experimental conditions. They were presented with the cover story that the study was about testing memory. The participants sat at one table with twelve cards lying on it (similar to those used in the popular memory cards game). They were informed that there were symbols on the other side of the cards and that each symbol had a pair. Next, one participant learned that she or he would be uncovering the cards one at a time in any order (agent condition), and the second participant learned that she or he would be looking at the cards that were being uncovered by the other participant (recipient condition). Both of the participants were asked to try to memorize the location of each symbol, and they were told there would be a recall test at the end of the study. They were asked not to communicate for the entire procedure.

We expected that in this interaction, the uncovering of the cards would result in taking the agent perspective because one performs and focuses on an action over which one has control (i.e., one decides on the order of cards to be uncovered and the time at which each card will be uncovered). Conversely, we expected that looking at the cards that were being uncovered by the second participant would result in taking the recipient perspective because one experiences the consequences of the agent’s actions, which involves the need to memorize the cards that are being managed by someone else and over which one has no control.

#### Procedure

The design was between subjects. After the manipulation, the participants were asked to complete the questionnaires, which were allegedly not related to the memory task. We used the same tools as in Experiment 1: the State Self-Esteem Scale (*α* = .81), the self-ascription of eight agentic (*α* = .85) and eight communal (*α* = .84) traits, and a manipulation check (*α* = .70). At the end, the participants reported their age and gender. The participants were informed that there would be no recall test and debriefed.

### Results

The analysis of the manipulation check items indicated that the manipulation was successful. The participants in the agent perspective condition agreed with the statements that during the study they had a sense of influence, they had control over the situation, they were not reliant on the other participant, and they were not passive to a greater extent than the participants in the recipient perspective condition did, *t*(45*)* = 5.76, *p* = .000, *d* = 1.67 (one-sided).

The moderation model, in which we verified whether taking the agent-recipient perspective moderates the importance of self-ascribed agency for self-esteem was again not significant, *b* = 0.09, 95% CI = [−0.16, 0.34], *t*(43) = 0.71, *p* = .482. Self-ascribed agency was significantly related to self-esteem among the agents, *b* = 0.23, 95% CI = [0.06, 0.40], *t*(43) = 2.73, *p* = .009 and not among the recipients, *b* = 0.14, 95% CI = [-0.04, 0.33], *t*(43) = 1.55, *p* = .129. Nevertheless, these two conditional effects are not significantly different, as there was no moderation effect.

The second hypothesized moderation model with self-ascribed communion traits as a predictor was also not significant, *b* = 0.01, 95% CI = [−0.31, 0.34], *t*(43) = 0.09, *p* = .930. Self-ascribed communal traits were not significantly related to self-esteem among the agents, *b* = -0.06, 95% CI = [-0.32, 0.20], *t*(43) = 0.43, *p* = .668, or among the recipients, *b* = -0.07, 95% CI = [-0.26, 0.12], *t*(43) = 0.73, *p* = .471.

In summary, the patterns of the relationship between agency-communion and self-esteem were not significantly different between the participants in the agent and the recipient perspective conditions.

## Experiment 3

The first two studies found no effect of higher importance of agency for the agent’s self-esteem and higher importance of communion for the recipient’s self-esteem. In Experiment 3, we went beyond manipulating the different roles in the interaction. First, we used a manipulation that addressed the focus of attention (performance versus experience). Furthermore, to address potential critics (*bottom-up approach* [47,48]) that the mere intention to act is not sufficient to induce a sense of agency we have introduced a manipulation accompanied by an actual (motor) action that had an impact on a direct environment. Following this notion, in the agent perspective condition, we assigned the participants an effortful motor task that had a visible effect at the end.

### Method

#### Participants

The participants were sixty-five undergraduate native Polish students from SWPS University of Social Sciences and Humanities in Warsaw (*M*_age_ = 23.14, *SD* = 5.26, 50 females, 15 males). Six participants who disbelieved the cover story and three participants who failed to properly complete the manipulation task were excluded from the analysis.

#### Manipulation

In the agent perspective condition, the participants’ task was to pump up an inflatable chair with an air pump, and they were asked to focus on this action. This task embodied actual, not merely conceptual, agency because it demanded physical effort and had a visible effect in the end (a chair that is ready to sit in). In the recipient perspective condition, the participants were invited to sit in the inflated chair for five minutes and asked to focus on the stimulus that affected them and the sensations that they were experiencing. Importantly, the cover story explained also that the "action involving a chair" was not related to the next phase, and the only reason that the participants were asked to pump up the chair/sit in the chair was to ‘rest their mind’ before the main task.

We expected that the pumping of the chair would result in taking the agent perspective because that individual performs an actual, motor action and focuses on his or her own performance, whereas sitting in the chair and thinking about the sensations that were being experienced would result in taking the recipient perspective because that individual focuses on the stimulus that affected him or her.

#### Procedure

The design was between subjects. After the manipulation, the participants were asked to complete the questionnaires: the State Self-Esteem Scale (*α* = .89) and self-ratings on eight agentic (*α* = .81) and eight communal traits (*α* = .76). Next, the manipulation check was assessed with four items (*In the chair phase*, *I felt I had no control over the situation; In the chair phase*, *I had a capacity to act; In the chair phase*, *I focused on performing some action; In the chair phase*, *I focused on experiencing various sensations;* answered on a scale from 1 [*definitely no]*, to 7 [*definitely yes*]). At the end, the participants reported their age and gender. During a debriefing session, we asked the participants if they believed that the chair phase had no relationship with the further assessments and what they thought the aim of the study was.

## Results

The analysis of the manipulation check items indicated that, as expected, the participants in the recipient perspective condition felt that they had no control over the situation, *t*(54) = 1.68, *p* = .049 (one-sided), and focused on experiencing various sensations, *t*(54) = 2.40, *p* = .010 (one-sided), to a greater extent than the participants in the agent perspective condition did, whereas the latter participants focused more on performing an action, *t*(54) = 2.02, *p* = .025 (one-sided). The participants in both conditions did not differ in response to a question about whether they had the capacity to act, *t*(54) = 0.08, *p* = .470 (one-sided). It is likely that the expression ‘capacity to act’ was too vague.

Again, there was no interaction of self-ascribed agency and the agent-recipient-perspective on self-esteem, *b* = -0.18, 95% CI = [-0.47, 0.11], *t*(52) = 1.26, *p* = .212. Self-ascribed agentic traits were significantly related to self-esteem both among the agents, *b* = 0.26, 95% CI = [0.03, 0.49], *t*(52) = 2.30, *p* = .026 and the recipients, *b* = 0.44, 95% CI = [0.26, 0.62], *t*(52) = 4.94, *p* = .000.

The interaction of self-ascribed communion and the agent-recipient-perspective on self-esteem was also not significant, *b* = -0.40, 95% CI = [-085, 0.06], *t*(52) = 1.80, *p* = .086. Self-ascribed communal traits were significantly related to self-esteem among the recipients, *b* = 0.45, 95% CI = [0.11, 0.79], *t*(52) = 2.62, *p* = .011, and not among the agents, *b* = 0.06, 95% CI = [-0.24, 0.35], *t*(52) = 0.37, *p* = .710. Nevertheless, these two conditional effects are not significantly different, as there was no moderation effect.

In summary, in Experiment 3 taking the agent and recipient perspectives was not manipulated through the one-to-one interaction as before but taking the agent perspective was operationalized as focusing on a motor action and taking the recipient perspective was operationalized as focusing on an experience. As was the case in two former studies, we found no support for Hypothesis 1 about the interaction effect of self-ascribed agency and the agent-recipient perspectives on self-esteem. We also did not find support for Hypothesis 2 about the interaction effect of self-ascribed communion and the agent-recipient perspectives on self-esteem, however the interaction effect was close to being significant in the predicted direction. Thus, in the next studies we continued with the manipulations that address the focus of attention (performance versus experience), and not just different roles in the interaction.

## Experiment 4

In the prior three experiments, we manipulated taking the agent and recipient perspectives with what the participants were *doing* (planning a day versus observing, uncovering the cards versus observing, pumping up the chair versus sitting in the chair). In the next studies, the manipulations were based on memory recollection and priming rather than actual behaviors. We continued to assess self-esteem and self-rating on agency and communion. However, we have also decided to measure more carefully the sub-categories of communal traits (namely morality and sociability), as previous studies have shown that the morality dimension might have its own specifics [20]. Thus, we used an extended list of agentic and communal traits, which capture morality and sociability separately within the communion dimension.

### Method

#### Participants

The participants were seventy-three undergraduate native Polish students from SWPS University of Social Sciences and Humanities in Warsaw (*M*_age_ = 24.23, *SD* = 7.08, 58 females, 12 males, and 2 people who did not specify their sex).

#### Manipulation

We used mindset-priming manipulation (i.e., when a person is asked to solve a task or a question), which requires the adoption of a specific cognitive mindset or processing style or the use of a specific mode of thinking in an action (as in a deliberative vs. implemental mind-sets [49]).

Participants were told that the study was commissioned by an online dating service and they would be surveyed as potential users of such service. The participants were presented with three questions, which differed in both conditions. Hence, in contrast to the prior manipulations, we did not ask the participants to act as agents or recipients. However, the questions that they answered were construed in a way that required taking the agent or the recipient perspective. The questions in the agent perspective condition provoked the participants to focus on and think about their own actions and decisions, whereas the questions in the recipient perspective condition provoked the participants to focus on and think about others and their impact on one’s thoughts and feelings.

The questions in the agent perspective condition were as follows: *How would you make the decision on whether to sign up for our service*?*; How would you use our service*?*; In which activities would you engage*?, and *What would you do to take the greatest advantage of our service to increase the effectiveness of finding the perfect date*? In the recipient perspective condition, the questions were as follows: *Being a user of our service means that other people look at your profile*, *description*, *and photo*, *and based on this they formulate their impression of you*. *Describe how would you feel knowing that someone is looking at your profile and judging you*?*; What kind of reactions on your profile would you expect and how would you feel about that*?; and *What kind of thoughts and feelings would you experience as a user of our service*?

#### Procedure

This was an online study with a between-subject design. After the manipulation, the participants completed Rosenberg’s Self-Esteem Scale [39] that was modified so that the items addressed current, not stable, attitudes toward oneself, *α* = .81. The self-ratings on agency and communion were assessed with a list of 21traits [50], seven of which referred to agency (*α* = .87), seven to morality (e.g., *honest*, *just; α* = .77), and seven to sociability (e.g., *kind*, *friendly*; *α* = .85). The participants indicated the extent to which each trait could be used to describe them on a scale from 1 (*definitely not*) to 7 (*definitely yes*). At the end, the participants reported their age and gender.

### Results

There was no interaction of perspectives with self-ascribed agency on self-esteem, *b* = -0.01, 95% CI = [-0.49, 0.46], *t*(69) = 0.06, *p* = .950. Self-ascribed agency was significantly related to self-esteem both among the participants in the agent condition, *b* = 0.85, 95% CI = [0.46, 1.23], *t*(69) = 4.42, *p* = .000 and the participants in the recipient condition, *b* = 0.86, 95% CI = [0.58, 1.14], *t*(69) = 6.20, *p* = .000.

There was also no interaction of perspectives with self-ascribed morality on self-esteem, *b* = 0.22, 95% CI = [-0.61, 1.04], *t*(69) = 0.58, *p* = .600. Self-ascribed morality was significantly related to self-esteem both among the agents, *b* = 0.84, 95% CI = [0.30, 1.38], *t*(69) = 3.09, *p* = .003 and the recipients, *b* = 0.62, 95% CI = [0.00, 1.24], *t*(69) = 1.99, *p* = .050.

Finally, there was also no interaction of perspectives with self-ascribed sociability on self-esteem, *b* = 0.16, 95% CI = [-0.48, 0.80], *t*(69) = 0.49, *p* = .628. Self-ascribed sociability was significantly related to self-esteem both among the agents, *b* = 0.75, 95% CI = [0.28, 1.22], *t*(69) = 3.21, *p* = .002 and the recipients, *b* = 0.59, 95% CI = [0.15, 1.03], *t*(69) = 2.69, *p* = .009.

To summarize, again we found no support for the hypothesis about the higher importance of agency for the self-esteem of the agents than for the recipients or a higher importance of communion (morality and sociability) for the self-esteem of the recipients than for the agents. In the next two studies, we investigated the potential moderators which could interact with taking the agent and recipient perspectives.

## Experiment 5

The constructs of the agent and recipient perspectives are very broad, and this is an evident obstacle in studying them. Therefore, we focused on specifying some contextual factors that could interfere with the perspectives.

In Experiment 5 we additionally manipulated the valence of the event (that is, of the action that is taken by the agent and experienced by the recipient). It appears likely that the self-perception processes might be affected by whether one did something good or something bad and whether one experienced someone else doing something good or something bad. We aimed to verify that the null results of the previous experiments were not due to interference with the valence effects. We did not make any specific predictions about the effect of the valence of the event on the hypothesized model, so in this regard the analysis was exploratory.

In Experiment 5, the studied sample was no longer comprised of students. It was a nationalwide sample that was recruited by a research agency.

### Method

#### Participants

The participants were 290 Polish respondents who were recruited from a nationwide sample (*M*_age_ = 34.51, *SD* = 11.9, 170 females, 118 males, and 2 persons who did not specify their sex; ranging from the age of 16 to 72).

#### Manipulation

We used an episodic recall manipulation. The participants were asked to recall and describe an event from their life when someone did something good or bad to someone else. There were four conditions: agent of a good action, agent of a bad action, recipient of a good action, and recipient of a bad action. The participants were presented with the following instruction: *Please recall an event when you (somebody) did something good/bad to somebody (you)*. *It is supposed to be an event in which you (somebody) were/was*, e.g., *honest*, *helpful*, *fair*, *supportive/dishonest*, *unhelpful*, *unfair*, *unsupportive to somebody (you)*. *It occurs to all of us to do (experience) something good/bad*, *so please try to recall such a situation and describe it from your own perspective*.

#### Procedure

This was an online study. The design was between subjects. After the manipulation, the participants were asked to complete the questionnaires while keeping the recalled event in mind. The self-ratings on agency and communion were again assessed with the list of 21traits (referring to agency, *α* = .89, morality, *α* = .93, and sociability, *α* = .93). This time, self-esteem was assessed with a single-item scale (see [40]): *Overall*, *I have a positive attitude toward myself* (responses on a scale from 1 (*definitely no*) to 7 (*definitely yes*)). At the end, the manipulation check was assessed with four items (*I described an event when somebody did something to me; I described an event when I did something to somebody; Most of all*, *I described what I did; Most of all*, *I described what I experienced;* answered on a scale from 1 [*definitely not]*, to 7 [*definitely yes*]). The participants also reported their age and gender.

### Results

The analysis of the manipulation check items indicated that, as expected, the participants in the agent perspective condition, compared to those in the recipient perspective condition, agreed more that they described an event when they did something to someone, *t*(288) = 17.93, *p* = .000 (one-sided), and described what they did, *t*(288) = 9.70, *p* = .000 (one-sided), whereas the participants in the recipient perspective condition, compared to those in the agent perspective condition, agreed more that they described an event when someone did something to them, *t*(288) = 14.76, *p* = .000 (one-sided) and described what they experienced, *t*(288) = 4.38, *p* = .000 (one-sided).

Next, we tested the moderated moderation models (Model 3 [42]) in which the interaction between the agent-recipient perspective (M) and the valence of the event (W) moderated the relationship between self-ascribed agency-communion (X) and self-esteem (Y). The analysis revealed there was no three-way interaction of the perspectives and the valence of the event with self-ascribed agency on self-esteem, *b* = -0.11, 95% CI = [-0.78, 0.56], *t*(282) = 0.32, *p* = .747. Self-ascribed agency was significantly related to self-esteem in all four conditions: agent of a good action, *b* = 0.42, 95% CI = [0.02, .81], *t*(282) = 2.05, *p* = .041, agent of a bad action, *b* = 0.69, 95% CI = [0.43, .94], *t*(282) = 5.29, *p* = .000, recipient of a good action, *b* = 0.57, 95% CI = [0.23, .91], *t*(282) = 3.29, *p* = .001, and recipient of a bad action, *b* = 0.73, 95% CI = [0.41, 1.06], *t*(282) = 4.44, *p* = .000.

The three-way interaction of the perspectives and the valence of the event with self-ascribed morality on self-esteem also was not significant, *b* = -0.23, 95% CI = [-0.95, 0.49], *t*(282) = 0.63, *p* = .527. Self-ascribed morality was significantly related to self-esteem in all four conditions: agent of a good action, *b* = 0.59, 95% CI = [0.18, 1.00], *t*(282) = 2.82, *p* = .005, agents of a bad action, *b* = 0.75, 95% CI = [0.56, 0.95], *t*(282) = 3.40, *p* = .001, recipient of a good action, *b* = 0.57, 95% CI = [0.09, 1.05], *t*(282) = 2.35, *p* = .019, and recipient of a bad action, *b* = 0.50, 95% CI = [0.21, 0.79], *t*(282) = 3.40, *p* = .001.

We found a significant three-way interaction of the perspectives and the valence of the event with self-ascribed sociability on self-esteem, *b* = -0.72, 95% CI = [-1.37, -0.07], *t*(282) = 2.18, *p* = .031. Self-ascribed sociability was significantly related to self-esteem in three of the four conditions: recipients of a good action, *b* = 0.75, 95% CI = [0.35, 1.14], *t*(282) = 3.74, *p* = .000, recipients of a bad action, *b* = 0.37, 95% CI = [0.09, .65], *t*(282) = 2.56, *p* = .011, and agent of a bad action, *b* = 0.70, 95% CI = [0.50, .91], *t*(282) = 6.68, *p* = .000. There was not a significant relationship between self-ascribed sociability and self-esteem in only one condition–agent of a good action, *b* = 0.36, 95% CI = [-0.03, .74], *t*(282) = 1.83, *p* = .069.

In summary, we did not find the predicted stronger association between self-ascribed agency and self-esteem in the agent than the recipient condition, and the stronger association between self-ascribed morality and self-esteem in the recipient than the agent condition, even when the valence of the event was included in the model as a potential second moderator. We found that self-ascribed sociability was significantly related to self-esteem in both variants of the recipient perspective condition, but only in one of two variants of the agent perspective condition, which could be considered as a little support for Hypothesis 2, but it also suggests that the effect of perspectives on sociability and self-esteem might interact with some contextual aspects.

## Experiment 6

In Experiment 6, we combined the tested model with one additional variable–status. We reasoned that in everyday life, taking the agent perspective often coincides with high status, while taking the recipient perspective coincides with low status. For example, one may argue that the dyadic manipulations we used in Experiments 1 and 2 could also be a manipulation of subjective status. In Experiment 6 we attempt to disentangle these two constructs by manipulating both perspective and status.

There is a general consensus among researchers that the subjective status has a significant impact on self-perception, is related to and affects self-esteem [51–53]. It is also related to agency and communion. Agency is often relatively heightened when individual’s role is awarded with high status [54,55]. Success is known to increase levels of self-ascribed agency. Abele, Rupprecht & Wojciszke [56] found that induction of success significantly improved level of declared agency (but not communion). Indeed, successful individuals are often higher in social hierarchy (upper class) and are expected to act in a competitive manner (expressing agentic qualities), while people from lower class are often dependent on each other (expressing communal qualities; c.f. [18]). Relatedly, Rucker, Galinsky and Magee [57] have formulated their model arguing that a sense of advantage (e.g. in social hierarchy) orients individuals toward agency (resulting in being independent from others), while a sense of disadvantage orients people toward communion (resulting in relying on mutual aid from friends and families). In terms of goals to pursue, Aydin, Ullrich, Siem, Locke, & Shnabel [58] have experimentally demonstrated that participants who imagined interactions with higher class targets expressed significantly stronger agentic goals to pursue with that person compared to participants who imagined interactions with lower class targets. Consequently, participants who imagined interactions with lower class targets declared significantly higher communal goals compared to participants who imagined interacting with higher class actors.

Here we tested the effect of both subjective status and agent-recipient perspective on the relationship between agency-communion and self-esteem. With regard to the interaction between status and perspective within the tested model the study was exploratory.

### Method

#### Participants

The participants were 141 respondents who were recruited from a nationwide Polish sample (*M*_age_ = 30.96, *SD* = 10.51, 106 females, 35 males, ranging from the age of 16 to 64).

#### Manipulation

We used an episodic recall manipulation. The four conditions were agent with high status, agent with low status, recipient with high status and recipient with low status. The participants were presented with the following instructions: *Please recall and describe an event from your life according to the two following criteria*: *(1) You performed some action and focused on performing it as best you could (you were a subject or recipient of someone else’s action and you experienced the consequences of it); (2) In that situation*, *you were in a privileged/underprivileged position*, *which means that in some sense you had a higher/lower position than the other person in terms of power or status*. The instruction also specified the following: *The event you that recall must fulfill both criteria at once*. *Please recall this event as vividly as you can*, *think of how you felt then and describe that memory from your perspective*.

#### Procedure

It was an online study. The design was between subjects. After the manipulation, the participants completed the assessments: the State Self-Esteem Scale (*α* = .92), self-ratings on 21 traits (referring to agency, *α* = .82, morality, *α* = .82, and sociability, *α* = .83). Next, the manipulation check was assessed with four items (*Describing an event*, *I focused on somebody’s influence on me; Describing an event*, *I was thinking about the action that I was performing then; Most of all*, *I described what I did in that situation; Most of all*, *I described what I experienced in that situation;* answered on a scale from 1 [*definitely not*], to 7 [*definitely yes*]). The participants also reported their age and gender.

### Results

The analysis of the manipulation check items indicated that, as expected, the participants in the agent perspective condition, compared to those in the recipient perspective condition, agreed more that when describing an event, they were thinking about the action that they were performing then, *t*(139) = 4.86, *p* = .000 (one-sided), and described what they did in that situation, *t*(139) = 4.79, *p* = .000 (one-sided), whereas the participants in the recipient perspective condition, compared to those in the agent perspective condition, agreed more that when describing an event, they focused on someone’s influence on them, *t*(139) = 3.08, *p* = .002 (one-sided), and described what they experienced in that situation, *t*(139) = 2.87, *p* = .003 (one-sided).

As in Study 5, we tested the moderated moderation models. The analysis revealed there was no three-way interaction of the perspectives and the status with self-ascribed agency on self-esteem, *b* = 0.44, 95% CI = [-0.28, 1.16], *t*(132) = 1.21, *p* = .228. Self-ascribed agency was significantly related to self-esteem in all four conditions: agent with high status, *b* = 0.89, 95% CI = [0.55, 1.24], *t*(132) = 5.13, *p* = .000, agent with low status, *b* = 0.57, 95% CI = [0.12, 1.02], *t*(132) = 2.49, *p* = .014, recipient with high status, *b* = 0.68, 95% CI = [0.33, 1.03], *t*(132) = 3.84, *p* = .000, and recipient with low status, *b* = 0.79, 95% CI = [0.52, 1.07], *t*(132) = 5.65, *p* = .000.

The three-way interaction of the perspectives and the status with self-ascribed morality on self-esteem was also not significant, *b* = 0.77, 95% CI = [-0.20, 1.74], *t*(132) = 1.56, *p* = .121. Self-ascribed morality was significantly related to self-esteem in two of the four conditions–agent with high status, *b* = 0.81, 95% CI = [0.37, 1.25], *t*(132) = 3.65, *p* = .000 and recipient with low status, *b* = 0.65, 95% CI = [0.32, 0.97], *t*(132) = 3.91, *p* = .000, and was not significantly related to self-esteem in two other conditions–agents with low status, *b* = 0.27, 95% CI = [-0.32, 0.85], *t*(132) = 0.90, *p* = .454 and recipients with high status, *b* = 0.42, 95% CI = [-0.13, 0.97], *t*(132) = 1.50, *p* = .137, nevertheless the effects were not significantly different between all four conditions, as there was no interaction effect.

The three-way interaction of the perspectives and the status with self-ascribed sociability on self-esteem was also not significant, *b* = 0.54, 95% CI = [-0.30, 1.38], *t*(132) = 1.28, *p* = .203. The same as in case of self-ascribed morality, self-ascribed sociability was significantly related to self-esteem in two of the four conditions–agent with high status, *b* = 0.59, 95% CI = [0.19, 0.99], *t*(132) = 2.92, *p* = .004 and recipient with low status, *b* = 0.49, 95% CI = [0.08, 0.89], *t*(132) = 2.36, *p* = .020, and was not significantly related to self-esteem in two other conditions–recipient with high status, *b* = 0.37, 95% CI = [-0.07, 0.82], *t*(132) = 1.66, *p* = .099 and agent with low status, *b* = 0.16, 95% CI = [-0.26, 0.58], *t*(132) = 0.75, *p* = .454. Again, please note that the interaction effect was not significant.

In summary, we found no support for the hypotheses derived from the Dual Perspective Model. Self-ascribed agency-communion was related to self-esteem with no significantly different strength in the agent and recipient conditions, independent of the status. The results of the simple slopes might suggest there are some differences with regard to self-ascribed communion, which seems to be more strongly related to self-esteem in some variants of the perspectives and status, however the differences were insignificant.

## Meta-analysis

We integrated the results of all of the experiments by conducting a meta-analysis. In the Comprehensive Meta-Analysis software, we input moderation effect size coefficients (weighted by the sample size) to estimate the moderation effect of the perspectives on the relationship between agency-communion and self-esteem. In Experiment 4, 5 and 6, we aggregated the morality and sociability subscales into a one index of self-ascribed communion. In Experiment 5 and 6, that included additional conditions (event valence and status) we computed a single effect size comparing the collapsed agent conditions with the collapsed recipient conditions. According to Cumming’s recommendation [59], we used the more conservative random effects model.

**Fig 1:**
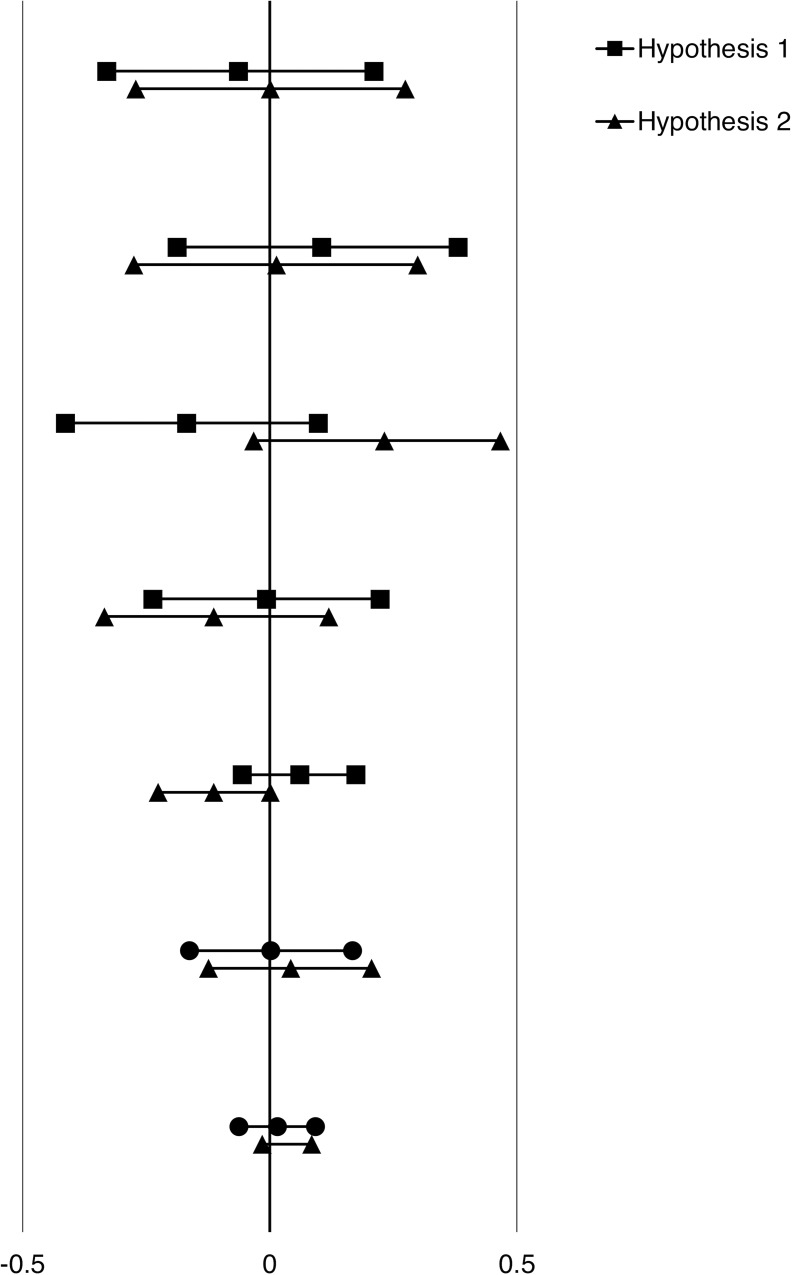
Forest plot showing effect sizes and confidence intervals for the Hypothesis 1 and Hypothesis 2.

The meta-analysis showed that self-ascribed agency was not more strongly related to self-esteem in the agent than in the recipient perspective, *r* = .02, 95% CI = [-0.06–0.09], *p* = .709, and that self-ascribed communion was not more strongly related to self-esteem in the recipient than in the agent perspective, *r* = -.02, 95% CI = [-0.12–0.08], *p* = .756 (Table 1, Fig 1). Agency was positively related to self-esteem both among the agents, *r* = .51, 95% CI = [0.46–0.59], *p =* .000 and the recipients, *r* = .58, 95% CI = [0.43–0.70], *p =* .000. Communion was also positively related to self-esteem both among the agents, *r* = .38, 95%CI = [0.13–0.59], *p* = .004 and the recipients, *r* = .38, 95%CI = [0.25–0.3649], *p =* .000.

**Table 1:** Studies included in the meta-analysis, manipulations, sample sizes, and effect sizes.

			Hypothesis 1^a^	Hypothesis 2^b^
Studies	Experimental manipulation	*N*	*r*	*r*
Experiment 1	*agent*: planning a day *recipient*: has their day being planned by someone else	52	-0.06	0.00
Experiment 2	*agent*: uncovering the cards *recipient*: looking at cards being uncovered by someone else	47	0.11	0.01
Experiment 3	*agent*: pumping up a chair and focusing on own performance *recipient*: sitting in a chair and focusing on own feelings	56	-0.17	0.23
Experiment 4	*agent*: considering online dating from agent perspective *recipient*: considering online dating from recipient perspective	73	-0.01	-0.11
Experiment 5^c^	*agent*: recalling an even of being an agent *recipient*: recalling an event of being a recipient	290	0.06	-0.11
Experiment 6^c^	*agent*: recalling an even of being an agent *recipient*: recalling an event of being a recipient	141	0.02	0.04
			Total .02	Total -.02

Note.

^a^Hypothesis 1 = Higher correlation of agency and self-esteem in agent than in recipient condition. Positive r*s* indicate higher correlation of agency with self-esteem in the agent than in the recipient condition.

^b^Hypothesis 2 = Higher correlation of communion and self-esteem in recipient than in agent condition. Positive r*s* indicate higher correlation of communion with self-esteem in the recipient than in the agent condition.

^c^Experiment 5 and 6 included additional conditions (event valence and status). We computed a single effect size comparing the collapsed agent conditions with the collapsed recipient conditions.

We also conducted additional analyses, which could be relevant in terms of the alternative explanations of the obtained null results. First, we tested if the manipulated agent-recipient perspectives affected the participants’ self-ascribed agentic and communal traits, as well as participants’ self-esteem. Meta-analysis addressing this question showed that there were no differences in self-ascribed agency, *d* = .11, 95%CI = [-0.05–0.26], *p* = .177, communion, *d* = .14, 95%CI = [-0.10–0.39], *p* = .257, and self-esteem, *d* = .32, 95%CI = [-0.17–0.81], *p* = .198, between the conditions (for descriptive statistics and differences go to S1 Table). We also tested our hypotheses in the regression models including self-ascribed agentic and communal traits, as well as participants’ gender together within one model. In all studies, adding gender to the model did not alter the results (S2 Table).

## Discussion

Across six experiments we found no support for the hypothesis that the relationship between self-ascribed agency-communion and self-esteem could be modified by the perspective one takes–the perspective of agent vs. the perspective of recipient. Our results provide novel experimental evidence that a temporary role or mindset had no substantial role in the link between the Big Two and self-esteem, although this possibility has been suggested by theorists (Dual Perspective Model [1,36]).

To investigate the effect of taking the agent-recipient perspectives on the importance of agency and communion for self-esteem, we conducted six experiments. We used multiple operationalizations of independent and dependent variables. The manipulations included behavioral manipulations, priming, and episodic recall. The main dependent variable, self-esteem, was assessed with three common measures of self-esteem. The samples comprised of students (Experiments 1–4) as well as nationalwide samples (Experiments 5 and 6). In addition to testing the main hypothesis, we tested potential moderators (the event valence and status). The results did not support the hypothesis that was derived from the Dual Perspective Model. In none of the six experiments self-ascribed agency was more strongly related to self-esteem in the agent than the recipient perspective, nor was self-ascribed communion more strongly related to self-esteem in the recipient than the agent perspective. Altogether, the meta-analysis indicated no effect on the different importance of agency and communion for self-esteem depending on the situational perspective taken.

### Concluding remarks on the null findings

Building on the Dual Perspective Model we expected that the agent-recipient perspectives would have an impact on agency-communion relatedness to self-esteem. However, the results of our studies rather imply that the relationship between self-ascribed agency-communion and feelings of self-worth is not modified under the influence of one’s temporary role or mindset.

Because we did not find the hypothesized differences between the experimental conditions, a question remains as to whether the manipulations that we used were successful. The manipulation check was assessed through self-report items, and the participants’ responses each time confirmed the predicted differences between the conditions. Unfortunately, such measures always bring a risk of demand characteristics. Nevertheless, we find it unlikely that our six manipulations did not induce the agent and the recipient perspectives. The operationalizations of taking the agent perspective were designed with respect to various definitions of agency that are present in the literature: by having the intention to act (e.g., when the participants recalled an event when they had a sense of doing something to somebody), by a motor experience of action (e.g., when the participants were pumping up the inflatable chair) and by having a choice (e.g., when the participants were scheduling a day or uncovering the cards). Similarly, taking the recipient perspective was induced in several various ways: by being passive during the other person’s actions (e.g., when the participants were only observing someone else scheduling a day for them or someone else uncovering the card that they needed to memorize), by being the subject of others’ actions (e.g., when the participants recalled an event when someone did something to them) and by concentrating on experience and receiving (e.g., when the participants who were sitting in a chair were monitoring the sensations that affected them or when they analyzed being judged and evaluated as users of an online dating service). Given that similar manipulations of various mindsets were commonly used in experimental psychology (e.g., deliberative/implemental or promotion/prevention mindset) and it enabled the observation of numerous, often subtle effects, we would expect the manipulations that we used to be successful and to enable the observation of the hypothesized effects, if they exist, especially in a meta-analysis.

In our studies, we intended for the agent and recipient perspectives to not be directly related to certain analogous constructs, such as power. However, we wonder if inducing these perspectives without the other states that naturally accompany them is actually valid. Taking the agent and recipient perspectives always arises from some context, such as social or professional roles. When this context is absent, one might take both perspectives, interchangeably focusing on different aspects of the situation or in accordance with the current motivations or one’s individual disposition in taking these two perspectives. Hence, we propose that the DPM could be investigated in the context of well-known dichotomies in terms of power, status, control, wealth, or social class. Finding the common pattern, such as a higher importance of agency among those who are in positions of power, possessing high status, leaders, speakers, or the rich and a higher importance of communion among those who are in positions of low power, possessing low status, followers, listeners, or the poor, would allow us to explain this common pattern with a meta-construct in terms of the taking of the agent and recipient perspectives.

A good theory is characterized as being internally consistent, logical and testable and by having simple predictions, the ability to explain many phenomena and to generate new ideas [60]. The DPM appears to meet all of these criteria. However, although a good theory should be broad, it cannot be too abstract [61] because it loses explanatory power. Reducing all of the complexity of social interactions to only two perspectives while neglecting specific contexts and separating it from other relevant constructs could explain why we failed to find differences between the perspectives. In this sense, the broadness of the DPM appeared to be its weakness. However, if the DPM’s hypotheses could provide support in research on classic dichotomies of the social cognition literature (such as leader-follower, speaker-listener, perpetrator-victim), the broadness of the DPM would be its main advantage. We suggest that this is a promising direction for further research on the DPM.

### Theoretical implications

It has been shown in numerous previous studies that agentic content is especially important in self-perception and the communal content is especially important in the perception of others [7]. The Dual Perspective Model extends this observation with the idea that in the agent perspective (compared to the recipient perspective) the agency dimension receives greater weight in both self-perception and the perception of others, while in the recipient perspective (compared to the agent perspective) the communion dimension receives greater weight in both self-perception and the perception of others. However, the present studies did not confirm such theorizing within the context of self-perception. They rather imply that the relationship between self-ascribed agency-communion and feelings of self-worth is not modified under the influence of one’s temporary role or mindset. Besides showing no evidence for the novel hypothesis on agency and communion in self-perception, our results replicate the previous findings. They show that agency relatedness to self-esteem is stable and prevalent (it occurred in all studies), while the relationship between communion and self-esteem although being in most studies, in some conditions did not occur at all and it was less stable and significantly weaker, *r* = .24 [0.13–0.35], *p* = .000 than in the case of agency, *r* = .45 [0.31–.0.57] *p* = .000; *Z* = 4.35, *p* = .000 (metanalytical overall effect).

The Dual Perspective Model is a broad theoretical concept that addresses the objective of powerful theorizing [62] by offering an integration of the previous findings on asymmetrical social relations and the Big Two in social cognition research. We believe that verifying such theories and reporting these verifications, even when they present null results, is important with regard to the integrity of the social cognition field and the self-correcting principle.

Our studies may also add to the discussion on what defines agency and how it can be induced in experimental studies. As for the definition of agency, Gallagher [63] argues that the sense of agency arises from an intention to act and can be operationalized through the personal belief that one did something or desired to do something. However, some researchers claim that the sense of agency might be induced without an intention of doing something, and they reason that it occurs on the level of neural processes that are triggered by motor actions [47,48,64,65]. Social psychology researchers usually define the sense of agency in terms of a sense of control over one’s own actions and, less frequently, over others [66]. Hence, the sense of agency is viewed as a state that is equivalent to a more dispositional sense of personal control. Some researchers emphasize that the crucial ingredient of a sense of agency is having a choice, and therefore it is not possible to experience a sense of agency when someone else makes decisions for us [67,68].

Considering the contribution of the present studies to this discussion, we suggest that a sense of agency does not arise from objective characteristics of the position that one takes, one’s actions or obtained results; rather, it arises from an individual’s interpretation of one's position and the aspects of the situation upon which one focuses. In one of our studies, the participants were asked to recall and describe an event in which they were either agents or recipients. Interestingly, some of the participants, despite being assigned to different conditions, described identical situations. For example, two participants described passing a driving license exam. The one who was assigned to the agent condition described performing driving maneuvers and a feeling of reaching a personal goal of becoming a driver, and the one who was assigned to the recipient condition described being dependent on the examiner and a feeling of being observed and evaluated the whole time. This example shows that it is not the action that is taken but rather the mindset that affects the sense of agency. Thus, we recommend that researchers who aim to experimentally manipulate the sense of agency move past just assigning roles for participants but also target the participants’ focus of attention on agentic or non-agentic aspects of the action.

### Limitations

A limitation of the present work is the small sample sizes, especially in Experiments 1, 2 and 3. These studies were conducted a few years ago when the smaller samples were much more common. Even so, we decided to report them, as in our opinion they enrich the paper with regard to diversified manipulations and their results strengthen the point of no support for the hypotheses tested. To address this problem and be able to include the underpowered studies in the present work, we integrated all the results within the meta-analysis. Nevertheless, we point out that the results of the underpowered studies must be interpreted with caution.

The manipulation check revealed a relatively weaker strength of the priming manipulations than the behavioral ones. We verified that the results for the hypothesized models are not different between the studies with behavioral and priming manipulations. Nevertheless, it seems that priming compared to behavioral manipulations had less impact, e.g., on participants’ self-esteem. These results contribute to the ongoing discussion about the effectiveness of social priming (e.g., [69].

Yet another limitation is that we used the explicit measures of self-esteem only. Although the previous studies showed that the agency-over-communion effect in self-esteem occurred even when self-esteem was measured implicitly as a preference for own initials [3], the fact that we did not include implicit measures limits the conclusions that can be drawn from the present project.

### Further steps

We found that the strength of the relationship between agency-communion and self-esteem did not differ in the agent and the recipient perspective. However, one may notice that the effect sizes for this effect differed across the experiments. This can inspire further studies about the moderators of the agency-communion importance for self-esteem. For example, although self-ascribed communion was significantly related to self-esteem in most of the experiments, there was no significant relationship between communion and self-esteem in Experiment 2, the only one that was set up in the context of competition (the memory test). Accordingly, we suggest that examining the meaning of various environments and situational demands, rather than of the individual’s mindset, could be a promising direction of further research on this topic.

In Experiment 3, self-ascribed communion was not related to self-esteem among those who were pumping up the chair, but it was among those sitting in the chair. We wonder if there is a relationship between the cognitive load and the agency-communion effect on self-esteem. Maybe interfering one’s self-esteem from own communion could be a more controlled process, thus when the cognitive resources are limited (remembering the cards in Experiment 2, focusing on an effortful motor task in Experiment 3) only the perception of own agency affects self-esteem.

### Conclusion

To conclude, the present research found that taking the agent versus the recipient perspective had no effect on the importance of agency and communion content for self-esteem. Our studies suggest that when in a temporary context and not combined with other related constructs, taking the agent and recipient perspectives has no substantial influence on what matters for one’s self-esteem. The relationship between self-perceived agency-communion and self-esteem seems to be considerably stable and independent of one’s temporary mindset

Although our results did not support the Dual Perspective Model hypotheses about the increased importance of agency in the agent perspective and the increased importance of communion in the recipient perspective in the context of self-evaluation, we believe DPM is a promising idea that demands further exploration in other context. When we consider any social interaction, we can identify the agent and the recipient perspective. It is the most basic and simple distinction of the social world, and it appears to be inevitable that such different perspectives (when we give versus receive, speak versus listen, touch versus being touched) entail different psychological functioning or mental states. The Dual Perspective Model generates many other hypotheses with regard not only to cognitive but also to affective, motivational and social aspects. We hope that our work, although it did not support some of the DPM hypotheses, will not diminish researchers’ interest in the investigation of this novel idea but rather that it will facilitate the recognition of the limitations of studying this phenomenon and provide direction for the development of the DPM and its methodology.

## Supporting information

S1 Table Descriptive statistics and differences in state self-esteem, self-ascribed agentic and communal traits between agent and recipient conditions in experiments.1,2,3,4,5 and 6. In Experiments 5 and 6 means that do not share the same letter within one variable are significantly different at the *p* < .05. * *p* < .05; ** *p* < .01; *** *p* < .01
(DOCX)

S2 Table Regression results using self esteem as the criterion presented separately for each study (1–6). *b* represents unstandardized regression weights. *beta* indicates the standardized regression weights. *LL* and *UL* indicate the lower and upper limits of a confidence interval, respectively. * indicates *p* < .05. ** indicates *p* < .01.
(DOCX)
