# Supplementary material for: Manipulated taking the agent versus the recipient perspective seems not to affect the relationship between agency-communion and self-esteem: A small-scale meta-analysis
Source: PLoS One. 2019 Feb 28;14(2):e0213183. doi: 10.1371/journal.pone.0213183 (PMC6394982; doi:10.1371/journal.pone.0213183)
Supplement: S1 Table — In Experiments 5 and 6 means that do not share the same letter within one variable are significantly different at the p < .05. * p < .05; ** p < .01; *** p < .01 (DOCX) [file pone.0213183.s001.docx]

**S1 Table. Descriptive Statistics and Differences in State Self-Esteem, Self-Ascribed Agentic and Communal Traits between Agent and Recipient Conditions in Experiments.1,2,3,4,5 and 6.**

|  | Agent | Recipient |  |  |
| --- | --- | --- | --- | --- |
|  | *M* (*SD*) | *M* (*SD*) | *t* |  |
| Experiment 1 |  |  |  |  |
| State self-esteem (1-5; Heatherton & Polivy, 1991) | 4.26 (0.37) | 3.71 (0.48) | 4.53*** |  |
| Self-ascribed agentic traits (1-7) | 5.21 (0.69) | 4.68 (1.14) | 1.95* |  |
| Self-ascribed communal traits (1-7) | 5.29 (0.85) | 5.51 (0.98) | 0.86 |  |
| Experiment 2 |  |  |  |  |
| State self-esteem (1-5; Heatherton & Polivy, 1991) | 4.07 (0.46) | 3.82 (0.42) | 1.92* |  |
| Self-ascribed agentic traits (1-7) | 5.17 (1.03) | 5.11 (0.93) | 0.21 |  |
| Self-ascribed communal traits (1-7) | 4.87 (0.72) | 5.37 (0.99) | 1.98 |  |
| Experiment 3 |  |  |  |  |
| State self-esteem (1-5; Heatherton & Polivy, 1991) | 3.87 (0.45) | 3.78 (0.70) | 0.59 |  |
| Self-ascribed agentic traits (1-7) | 1.45 (0.83) | 1.24 (1.02) | 0.85 |  |
| Self-ascribed communal traits (1-7) | 1.94 (0.76) | 2.07 (0.62) | 0.72 |  |
| Experiment 4 |  |  |  |  |
| State self-esteem (1-7; modified SES Rosenberg, 1965) | 5.37 (1.18) | 5.37 (1.22) | 0.03 |  |
| Self-ascribed agentic traits (1-7) | 5.46 (0.77) | 5.25 (1.10) | 0.95 |  |
| Self-ascribed moral traits (1-7) | 5.73 (0.67) | 5.76 (0.61) | 0.17 |  |
| Self-ascribed sociability traits (1-7) | 5.85 (0.76) | 5.70 (0.84) | 0.79 |  |
| Experiment 5 |  |  |  |  |
| State self-esteem (1-5; single-item scale) |  |  |  |  |
| Good action | 5.79_a_ (1.03) | 5.51_a_ (1.33) | – | |
| Bad action | 3.80_b_ (1.88) | 4.72_c_ (1.70) | – | |
| Self-ascribed agentic traits (1-7) |  |  |  |  |
| Good action | 5.29_b_ (0.76) | 4.98_a_ (0.97) | – | |
| Bad action | 4.69_c_ (1.23) | 5.02_a_ (1.04) | – | |
| Self-ascribed moral traits (1-7) |  |  |  |  |
| Good action | 5.59_a_ (0.72) | 5.61_ab_ (0.67) | – | |
| Bad action | 4.34_c_ (1.55) | 5.45_b_ (1.14) | – | |
| Self-ascribed sociability traits (1-7) |  |  |  |  |
| Good action | 5.59_a_ (0.78) | 5.40_ab_ (0.84) | – | |
| Bad action | 4.34_c_ (1.50) | 5.08_b_ (1.03) | – | |
| Experiment 6 |  |  |  | |
| State self-esteem (1-5; Heatherton & Polivy, 1991) |  |  |  |  |
| High status | 4.62_a_ (1.08) | 4.91_a_ (0.82) | – | |
| Low status | 4.80_a_ (0.85) | 4.54_a_ (1.01) | – | |
| Self-ascribed agentic traits (1-7) |  |  |  |  |
| High status | 5.36_a_ (0.71) | 5.36_a_ (0.77) | – | |
| Low status | 5.31_a_ (0.69) | 5.22_a_ (0.86) | – | |
| Self-ascribed moral traits (1-7) |  |  |  |  |
| High status | 5.82_a_ (0.63) | 5.71_a_ (0.55) | – | |
| Low status | 5.85_a_ (0.61) | 5.67_a_ (0.83) | – | |
| Self-ascribed sociability traits (1-7) |  |  |  |  |
| High status | 5.70_a_ (0.79) | 5.61_a_ (0.72) | – | |
| Low status | 5.71_a_ (0.88) | 5.56_a_ (0.70) | – | |

*Note.* In Experiments 5 and 6 means that do not share the same letter within one variable are significantly different at the *p* < .05.

* *p* < .05; ** *p* < .01; *** *p* < .01
