# Supplementary material for: Manipulated taking the agent versus the recipient perspective seems not to affect the relationship between agency-communion and self-esteem: A small-scale meta-analysis
Source: PLoS One. 2019 Feb 28;14(2):e0213183. doi: 10.1371/journal.pone.0213183 (PMC6394982; doi:10.1371/journal.pone.0213183)
Supplement: S2 Table — b represents unstandardized regression weights. beta indicates the standardized regression weights. LL and UL indicate the lower and upper limits of a confidence interval, respectively. * indicates p < .05. ** indicates p < .01. (DOCX) [file pone.0213183.s002.docx]

**S2 Table. Regression Results Using Self Esteem as the Criterion Presented Separately for Each Study (1-6).**

Study 1

| Predictor | *b* | *b*  95% CI  [LL, UL] | *beta* | *beta*  95% CI  [LL, UL] | Fit | | Difference | | |  |  |
| --- | --- | --- | --- | --- | --- | --- | --- | --- | --- | --- | --- |
| (Intercept) | 2.29** | [1.46, 3.11] |  |  |  | |  | | |  |  |
| AGENCY | 0.26** | [0.13, 0.39] | 0.52 | [0.26, 0.77] |  | |  | | |  |  |
| COMMUNION | 0.07 | [-0.06, 0.21] | 0.14 | [-0.12, 0.39] |  | |  | | |  |  |
|  |  |  |  |  | *R^2^*  = .332** | |  | | |  |  |
|  |  |  |  |  | 95% CI[.11,.49] | |  | | |  |  |
|  |  |  |  |  |  | |  | | |  |  |
| (Intercept) | 2.14** | [1.43, 2.85] |  |  |  | |  | | |  |  |
| AGENCY | 0.18** | [0.07, 0.30] | 0.36 | [0.13, 0.59] |  | |  | | |  |  |
| COMMUNION | 0.13* | [0.01, 0.25] | 0.24 | [0.02, 0.47] |  | |  | | |  |  |
| PERSPECTIVE | 0.45** | [0.24, 0.67] | 0.46 | [0.24, 0.68] |  | |  | | |  |  |
|  |  |  |  |  | *R^2^*  = .520** | | Δ*R^2^*  = .188** | | |  |  |
|  |  |  |  |  | 95% CI[.28,.64] | | 95% CI[.03, .35] | | |  |  |
|  |  |  |  |  |  | |  | | |  |  |
| (Intercept) | 2.13** | [1.37, 2.90] |  |  |  | |  | | |  |  |
| AGENCY | 0.18** | [0.06, 0.30] | 0.36 | [0.12, 0.59] |  | |  | | |  |  |
| COMMUNION | 0.13* | [0.01, 0.26] | 0.24 | [0.02, 0.47] |  | |  | | |  |  |
| PERSPECTIVE | 0.45** | [0.23, 0.67] | 0.46 | [0.24, 0.69] |  | |  | | |  |  |
| SEX | 0.01 | [-0.23, 0.25] | 0.01 | [-0.20, 0.22] |  | |  | | |  |  |
|  |  |  |  |  | *R^2^*  = .520** | | Δ*R^2^*  = .000 | | |  |  |
|  |  |  |  |  | 95% CI[.26,.63] | | 95% CI[-.00, .00] | | |  |  |
|  |  |  |  |  |  | |  | | |  |  |
| (Intercept) | 2.41** | [1.47, 3.34] |  |  |  | |  | | |  |  |
| AGENCY | 0.22** | [0.05, 0.38] | 0.42 | [0.11, 0.74] |  | |  | | |  |  |
| COMMUNION | 0.06 | [-0.13, 0.25] | 0.11 | [-0.24, 0.45] |  | |  | | |  |  |
| PERSPECTIVE | -0.20 | [-1.88, 1.48] | -0.20 | [-1.93, 1.52] |  | |  | | |  |  |
| SEX | -0.01 | [-0.25, 0.24] | -0.01 | [-0.22, 0.21] |  | |  | | |  |  |
| COMMUNION * PERSPECTIVE | 0.14 | [-0.12, 0.40] | 0.79 | [-0.66, 2.23] |  | |  | | |  |  |
| AGENCY * PERSPECTIVE | -0.02 | [-0.29, 0.24] | -0.14 | [-1.60, 1.32] |  | |  | | |  |  |
|  |  |  |  |  | *R^2^*  = .533** | | Δ*R^2^*  = .013 | | |  |  |
|  |  |  |  |  | 95% CI[.24,.63] | | 95% CI[-.03, .06] | | |  |  |
|  |  |  |  |  |  |  | |  |  | |  |

Study 2

| Predictor | *b* | *b*  95% CI  [LL, UL] | *beta* | *beta*  95% CI  [LL, UL] | Fit | | Difference | | |  |  |
| --- | --- | --- | --- | --- | --- | --- | --- | --- | --- | --- | --- |
| (Intercept) | 3.42** | [2.17, 4.68] |  |  |  | |  | | |  |  |
| AGENCY | 0.18* | [0.04, 0.32] | 0.37 | [0.07, 0.66] |  | |  | | |  |  |
| COMMUNION | -0.08 | [-0.25, 0.09] | -0.13 | [-0.43, 0.16] |  | |  | | |  |  |
|  |  |  |  |  | *R^2^*  = .176* | |  | | |  |  |
|  |  |  |  |  | 95% CI[.00,.35] | |  | | |  |  |
|  |  |  |  |  |  | |  | | |  |  |
| (Intercept) | 3.00** | [1.71, 4.29] |  |  |  | |  | | |  |  |
| AGENCY | 0.17* | [0.04, 0.31] | 0.36 | [0.08, 0.65] |  | |  | | |  |  |
| COMMUNION | -0.02 | [-0.19, 0.16] | -0.03 | [-0.34, 0.27] |  | |  | | |  |  |
| PERSPECTIVE | 0.26 | [-0.01, 0.53] | 0.28 | [-0.01, 0.58] |  | |  | | |  |  |
|  |  |  |  |  | *R^2^*  = .246** | | Δ*R^2^*  = .070 | | |  |  |
|  |  |  |  |  | 95% CI[.02,.41] | | 95% CI[-.06, .20] | | |  |  |
|  |  |  |  |  |  | |  | | |  |  |
| (Intercept) | 2.65** | [1.36, 3.94] |  |  |  | |  | | |  |  |
| AGENCY | 0.14* | [0.00, 0.27] | 0.28 | [0.00, 0.57] |  | |  | | |  |  |
| COMMUNION | 0.00 | [-0.17, 0.17] | 0.01 | [-0.29, 0.30] |  | |  | | |  |  |
| PERSPECTIVE | 0.30* | [0.04, 0.57] | 0.33 | [0.04, 0.62] |  | |  | | |  |  |
| SEX | 0.27* | [0.01, 0.53] | 0.30 | [0.01, 0.58] |  | |  | | |  |  |
|  |  |  |  |  | *R^2^*  = .324** | | Δ*R^2^*  = .078* | | |  |  |
|  |  |  |  |  | 95% CI[.05,.47] | | 95% CI[-.05, .21] | | |  |  |
|  |  |  |  |  |  | |  | | |  |  |
| (Intercept) | 3.24** | [1.36, 5.12] |  |  |  | |  | | |  |  |
| AGENCY | 0.05 | [-0.15, 0.26] | 0.11 | [-0.31, 0.54] |  |  |  |  |  |  |  |
| COMMUNION | -0.03 | [-0.27, 0.21] | -0.05 | [-0.47, 0.37] |  |  |  |  |  |  |  |
| PERSPECTIVE | -0.74 | [-3.19, 1.72] | -0.81 | [-3.51, 1.89] |  |  |  |  |  |  |  |
| SEX | 0.27* | [0.01, 0.53] | 0.30 | [0.01, 0.58] |  |  |  |  |  |  |  |
| COMMUNION * PERSPECTIVE | 0.06 | [-0.28, 0.40] | 0.32 | [-1.54, 2.19] |  |  |  |  |  |  |  |
| AGENCY * PERSPECTIVE | 0.14 | [-0.12, 0.41] | 0.85 | [-0.73, 2.43] |  |  |  |  |  |  |  |
|  |  |  |  |  | *R^2^*  = .345* | | Δ*R^2^*  = .021 | | |  |  |
|  |  |  |  |  | 95% CI[.02,.46] | | 95% CI[-.05, .09] | | |  |  |
|  |  |  |  |  |  |  | |  |  | |  |

Study 3

| Predictor | *b* | *b*  95% CI  [LL, UL] | *beta* | *beta*  95% CI  [LL, UL] | Fit | | Difference | | |  |  |
| --- | --- | --- | --- | --- | --- | --- | --- | --- | --- | --- | --- |
| (Intercept) | 3.23** | [2.86, 3.61] |  |  |  | |  | | |  |  |
| AGENCY | 0.36** | [0.22, 0.50] | 0.57 | [0.35, 0.79] |  | |  | | |  |  |
| COMMUNION | 0.07 | [-0.12, 0.26] | 0.08 | [-0.14, 0.30] |  | |  | | |  |  |
|  |  |  |  |  | *R^2^*  = .364** | |  | | |  |  |
|  |  |  |  |  | 95% CI[.16,.50] | |  | | |  |  |
|  |  |  |  |  |  | |  | | |  |  |
| (Intercept) | 3.16** | [2.76, 3.57] |  |  |  | |  | | |  |  |
| AGENCY | 0.35** | [0.21, 0.49] | 0.56 | [0.34, 0.78] |  | |  | | |  |  |
| COMMUNION | 0.08 | [-0.11, 0.27] | 0.09 | [-0.13, 0.31] |  | |  | | |  |  |
| PERSPECTIVE | 0.12 | [-0.14, 0.37] | 0.10 | [-0.11, 0.30] |  | |  | | |  |  |
|  |  |  |  |  | *R^2^*  = .373** | | Δ*R^2^*  = .009 | | |  |  |
|  |  |  |  |  | 95% CI[.16,.50] | | 95% CI[-.03, .05] | | |  |  |
|  |  |  |  |  |  | |  | | |  |  |
| (Intercept) | 3.45** | [2.90, 4.01] |  |  |  | |  | | |  |  |
| AGENCY | 0.36** | [0.22, 0.50] | 0.57 | [0.35, 0.79] |  | |  | | |  |  |
| COMMUNION | 0.07 | [-0.12, 0.26] | 0.08 | [-0.14, 0.30] |  | |  | | |  |  |
| PERSPECTIVE | 0.12 | [-0.13, 0.37] | 0.10 | [-0.11, 0.30] |  | |  | | |  |  |
| SEX | -0.22 | [-0.52, 0.07] | -0.16 | [-0.36, 0.05] |  | |  | | |  |  |
|  |  |  |  |  | *R^2^*  = .397** | | Δ*R^2^*  = .024 | | |  |  |
|  |  |  |  |  | 95% CI[.17,.52] | | 95% CI[-.04, .08] | | |  |  |
|  |  |  |  |  |  | |  | | |  |  |
| (Intercept) | 3.21** | [2.47, 3.94] |  |  |  | |  | | |  |  |
| AGENCY | 0.39** | [0.19, 0.58] | 0.61 | [0.31, 0.92] |  | |  | | |  |  |
| COMMUNION | 0.17 | [-0.14, 0.48] | 0.20 | [-0.16, 0.56] |  | |  | | |  |  |
| PERSPECTIVE | 0.51 | [-0.28, 1.29] | 0.41 | [-0.23, 1.06] |  | |  | | |  |  |
| SEX | -0.22 | [-0.51, 0.08] | -0.15 | [-0.36, 0.05] |  | |  | | |  |  |
| COMMUNION * PERSPECTIVE | -0.15 | [-0.55, 0.24] | -0.28 | [-1.00, 0.43] |  | |  | | |  |  |
| AGENCY * PERSPECTIVE | -0.06 | [-0.34, 0.22] | -0.09 | [-0.55, 0.36] |  | |  | | |  |  |
|  |  |  |  |  | *R^2^*  = .409** | | Δ*R^2^*  = .012 | | |  |  |
|  |  |  |  |  | 95% CI[.15,.51] | | 95% CI[-.03, .05] | | |  |  |
|  |  |  |  |  |  |  | |  |  | |  |

Study 4

| Predictor | *b* | *b*  95% CI  [LL, UL] | *beta* | *beta*  95% CI  [LL, UL] | Fit | Difference |
| --- | --- | --- | --- | --- | --- | --- |
| (Intercept) | -0.56 | [-2.44, 1.32] |  |  |  |  |
| AGENCY | 0.72** | [0.47, 0.98] | 0.57 | [0.37, 0.77] |  |  |
| COMMUNION | 0.36 | [-0.02, 0.73] | 0.19 | [-0.01, 0.39] |  |  |
|  |  |  |  |  | *R^2^*  = .477** |  |
|  |  |  |  |  | 95% CI[.30,.59] |  |
|  |  |  |  |  |  |  |
| (Intercept) | -0.52 | [-2.40, 1.37] |  |  |  |  |
| AGENCY | 0.74** | [0.48, 0.99] | 0.58 | [0.38, 0.78] |  |  |
| COMMUNION | 0.35 | [-0.02, 0.73] | 0.19 | [-0.01, 0.39] |  |  |
| PERSPECTIVE | -0.18 | [-0.59, 0.23] | -0.08 | [-0.25, 0.10] |  |  |
|  |  |  |  |  | *R^2^*  = .483** | Δ*R^2^*  = .006 |
|  |  |  |  |  | 95% CI[.29,.59] | 95% CI[-.02, .03] |
|  |  |  |  |  |  |  |
| (Intercept) | -1.13 | [-3.26, 1.00] |  |  |  |  |
| AGENCY | 0.71** | [0.45, 0.97] | 0.56 | [0.36, 0.77] |  |  |
| COMMUNION | 0.43* | [0.04, 0.81] | 0.23 | [0.02, 0.44] |  |  |
| PERSPECTIVE | -0.17 | [-0.58, 0.24] | -0.07 | [-0.25, 0.10] |  |  |
| SEX | 0.27 | [-0.17, 0.71] | 0.11 | [-0.07, 0.29] |  |  |
|  |  |  |  |  | *R^2^*  = .494** | Δ*R^2^*  = .011 |
|  |  |  |  |  | 95% CI[.29,.60] | 95% CI[-.02, .05] |
|  |  |  |  |  |  |  |
| (Intercept) | 0.60 | [-2.17, 3.37] |  |  |  |  |
| AGENCY | 0.85** | [0.53, 1.18] | 0.68 | [0.42, 0.93] |  |  |
| COMMUNION | 0.01 | [-0.54, 0.56] | 0.00 | [-0.29, 0.30] |  |  |
| PERSPECTIVE | -3.18 | [-6.97, 0.61] | -1.35 | [-2.96, 0.26] |  |  |
| SEX | 0.19 | [-0.25, 0.63] | 0.08 | [-0.10, 0.26] |  |  |
| COMMUNION * PERSPECTIVE | 0.79* | [0.05, 1.53] | 1.96 | [0.12, 3.80] |  |  |
| AGENCY * PERSPECTIVE | -0.29 | [-0.81, 0.24] | -0.68 | [-1.91, 0.56] |  |  |
|  |  |  |  |  | *R^2^*  = .527** | Δ*R^2^*  = .032 |
|  |  |  |  |  | 95% CI[.31,.61] | 95% CI[-.02, .09] |
|  |  |  |  |  |  |  |

Study 5

| Predictor | *b* | *b*  95% CI  [LL, UL] | *beta* | *beta*  95% CI  [LL, UL] | Fit | | Difference | | |  |  |
| --- | --- | --- | --- | --- | --- | --- | --- | --- | --- | --- | --- |
| (Intercept) | -0.56 | [-2.44, 1.32] |  |  |  | |  | | |  |  |
| AGENCY | 0.72** | [0.47, 0.98] | 0.57 | [0.37, 0.77] |  | |  | | |  |  |
| COMMUNION | 0.36 | [-0.02, 0.73] | 0.19 | [-0.01, 0.39] |  | |  | | |  |  |
|  |  |  |  |  | *R^2^*  = .477** | |  | | |  |  |
|  |  |  |  |  | 95% CI[.30,.59] | |  | | |  |  |
|  |  |  |  |  |  | |  | | |  |  |
| (Intercept) | -0.52 | [-2.40, 1.37] |  |  |  | |  | | |  |  |
| AGENCY | 0.74** | [0.48, 0.99] | 0.58 | [0.38, 0.78] |  | |  | | |  |  |
| COMMUNION | 0.35 | [-0.02, 0.73] | 0.19 | [-0.01, 0.39] |  | |  | | |  |  |
| PERSPECTIVE | -0.18 | [-0.59, 0.23] | -0.08 | [-0.25, 0.10] |  | |  | | |  |  |
|  |  |  |  |  | *R^2^*  = .483** | | Δ*R^2^*  = .006 | | |  |  |
|  |  |  |  |  | 95% CI[.29,.59] | | 95% CI[-.02, .03] | | |  |  |
|  |  |  |  |  |  | |  | | |  |  |
| (Intercept) | -1.13 | [-3.26, 1.00] |  |  |  | |  | | |  |  |
| AGENCY | 0.71** | [0.45, 0.97] | 0.56 | [0.36, 0.77] |  | |  | | |  |  |
| COMMUNION | 0.43* | [0.04, 0.81] | 0.23 | [0.02, 0.44] |  | |  | | |  |  |
| PERSPECTIVE | -0.17 | [-0.58, 0.24] | -0.07 | [-0.25, 0.10] |  | |  | | |  |  |
| SEX | 0.27 | [-0.17, 0.71] | 0.11 | [-0.07, 0.29] |  | |  | | |  |  |
|  |  |  |  |  | *R^2^*  = .494** | | Δ*R^2^*  = .011 | | |  |  |
|  |  |  |  |  | 95% CI[.29,.60] | | 95% CI[-.02, .05] | | |  |  |
|  |  |  |  |  |  | |  | | |  |  |
| (Intercept) | 0.60 | [-2.17, 3.37] |  |  |  | |  | | |  |  |
| AGENCY | 0.85** | [0.53, 1.18] | 0.68 | [0.42, 0.93] |  | |  | | |  |  |
| COMMUNION | 0.01 | [-0.54, 0.56] | 0.00 | [-0.29, 0.30] |  | |  | | |  |  |
| PERSPECTIVE | -3.18 | [-6.97, 0.61] | -1.35 | [-2.96, 0.26] |  | |  | | |  |  |
| SEX | 0.19 | [-0.25, 0.63] | 0.08 | [-0.10, 0.26] |  | |  | | |  |  |
| COMMUNION * PERSPECTIVE | 0.79* | [0.05, 1.53] | 1.96 | [0.12, 3.80] |  | |  | | |  |  |
| AGENCY * PERSPECTIVE | -0.29 | [-0.81, 0.24] | -0.68 | [-1.91, 0.56] |  | |  | | |  |  |
|  |  |  |  |  | *R^2^*  = .527** | | Δ*R^2^*  = .032 | | |  |  |
|  |  |  |  |  | 95% CI[.31,.61] | | 95% CI[-.02, .09] | | |  |  |
|  |  |  |  |  |  |  | |  |  | |  |

Study 6

| Predictor | *b* | *b*  95% CI  [LL, UL] | *beta* | *beta*  95% CI  [LL, UL] | Fit | Difference |
| --- | --- | --- | --- | --- | --- | --- |
| (Intercept) | 0.67 | [-0.59, 1.94] |  |  |  |  |
| AGENCY | 0.76** | [0.51, 1.01] | 0.59 | [0.40, 0.79] |  |  |
| COMMUNION | -0.01 | [-0.30, 0.29] | -0.00 | [-0.20, 0.19] |  |  |
|  |  |  |  |  | *R^2^*  = .351** |  |
|  |  |  |  |  | 95% CI[.21,.46] |  |
|  |  |  |  |  |  |  |
| (Intercept) | 0.67 | [-0.60, 1.94] |  |  |  |  |
| AGENCY | 0.76** | [0.51, 1.01] | 0.59 | [0.40, 0.79] |  |  |
| COMMUNION | -0.00 | [-0.31, 0.30] | -0.00 | [-0.20, 0.20] |  |  |
| PERSPECTIVE | -0.00 | [-0.29, 0.28] | -0.00 | [-0.15, 0.14] |  |  |
|  |  |  |  |  | *R^2^*  = .351** | Δ*R^2^*  = .000 |
|  |  |  |  |  | 95% CI[.21,.45] | 95% CI[-.00, .00] |
|  |  |  |  |  |  |  |
| (Intercept) | 0.16 | [-1.24, 1.56] |  |  |  |  |
| AGENCY | 0.72** | [0.46, 0.98] | 0.56 | [0.36, 0.76] |  |  |
| COMMUNION | 0.06 | [-0.25, 0.38] | 0.04 | [-0.16, 0.25] |  |  |
| PERSPECTIVE | -0.06 | [-0.35, 0.23] | -0.03 | [-0.18, 0.12] |  |  |
| SEX | 0.28 | [-0.05, 0.61] | 0.13 | [-0.02, 0.28] |  |  |
|  |  |  |  |  | *R^2^*  = .365** | Δ*R^2^*  = .015 |
|  |  |  |  |  | 95% CI[.22,.46] | 95% CI[-.02, .05] |
|  |  |  |  |  |  |  |
| (Intercept) | 0.57 | [-1.33, 2.48] |  |  |  |  |
| AGENCY | 0.77** | [0.41, 1.14] | 0.61 | [0.32, 0.89] |  |  |
| COMMUNION | -0.06 | [-0.54, 0.42] | -0.04 | [-0.36, 0.28] |  |  |
| PERSPECTIVE | -0.84 | [-3.44, 1.76] | -0.43 | [-1.78, 0.91] |  |  |
| SEX | 0.29 | [-0.05, 0.63] | 0.13 | [-0.02, 0.29] |  |  |
| COMMUNION * PERSPECTIVE | 0.22 | [-0.40, 0.83] | 0.66 | [-1.22, 2.53] |  |  |
| AGENCY * PERSPECTIVE | -0.09 | [-0.60, 0.43] | -0.24 | [-1.69, 1.20] |  |  |
|  |  |  |  |  | *R^2^*  = .368** | Δ*R^2^*  = .003 |
|  |  |  |  |  | 95% CI[.20,.46] | 95% CI[-.01, .02] |
|  |  |  |  |  |  |  |

*Note.* *b* represents unstandardized regression weights. *beta* indicates the standardized regression weights. *LL* and *UL* indicate the lower and upper limits of a confidence interval, respectively.

* indicates *p* < .05. ** indicates *p* < .01.
